# The Challenges of O_2_ Detection in Biological Fluids: Classical Methods and Translation to Clinical Applications

**DOI:** 10.3390/ijms232415971

**Published:** 2022-12-15

**Authors:** Valentina Marassi, Stefano Giordani, Andjela Kurevija, Emilio Panetta, Barbara Roda, Nan Zhang, Andrea Azzolini, Sara Dolzani, Dmytro Manko, Pierluigi Reschiglian, Mauro Atti, Andrea Zattoni

**Affiliations:** 1 Department of Chemistry G. Ciamician, University of Bologna, 40126 Bologna, Italy; 2 byFlow srl, 40129 Bologna, Italy; 3 Aferetica srl, 40138 Bologna, Italy; 4 Dinamica Generale S.p.A., 46025 Poggio Rusco, Italy

**Keywords:** dissolved oxygen quantification, biological fluids, clinical applications, oxygen sensing, absolute and relative techniques, technical innovation in clinics

## Abstract

Dissolved oxygen (DO) is deeply involved in preserving the life of cellular tissues and human beings due to its key role in cellular metabolism: its alterations may reflect important pathophysiological conditions. DO levels are measured to identify pathological conditions, explain pathophysiological mechanisms, and monitor the efficacy of therapeutic approaches. This is particularly relevant when the measurements are performed in vivo but also in contexts where a variety of biological and synthetic media are used, such as ex vivo organ perfusion. A reliable measurement of medium oxygenation ensures a high-quality process. It is crucial to provide a high-accuracy, real-time method for DO quantification, which could be robust towards different medium compositions and temperatures. In fact, biological fluids and synthetic clinical fluids represent a challenging environment where DO interacts with various compounds and can change continuously and dynamically, and further precaution is needed to obtain reliable results. This study aims to present and discuss the main oxygen detection and quantification methods, focusing on the technical needs for their translation to clinical practice. Firstly, we resumed all the main methodologies and advancements concerning dissolved oxygen determination. After identifying the main groups of all the available techniques for DO sensing based on their mechanisms and applicability, we focused on transferring the most promising approaches to a clinical in vivo/ex vivo setting.

## 1. Introduction

Oxygen (O_2_) is one of the key molecules of life, playing a major role in cellular metabolism. O_2_ has a high redox potential making it an ideal electron acceptor and, therefore, a sink for the capture of energy for intracellular use [1]. To be exploited by living beings, oxygen is taken up reversibly from the atmosphere and transported to oxygen-depleted tissues, where it is stored until actual use.

Approximately 90 to 95% of the dissolved oxygen (DO) consumed by the body is utilized by mitochondria to supply cellular energy through respiration and oxidative phosphorylation [2,3]. Based on that, it is easy to understand how regulation of tissue oxygenation and maintenance of adequate O_2_ levels are fundamental requirements for a healthy organism. Consequently, DO levels represent a significative indicator to evaluate pathological conditions (such as abnormally low or high DO levels, hypoxia, and hyperoxia, respectively) and may explain pathophysiological mechanisms and monitor the effects of therapeutic treatments [4].

A series of methods for determining DO in the various aqueous and biological matrixes have been developed. The main ones include iodometric titration, electrochemical methods, and optical methods. However, despite its profound biological and clinical importance, a limited number of effective methods exist for quantifying DO in its physiological settings, and real-time measurements are not always available. Therefore, it is necessary to improve the ability to quantify oxygen levels to further study its profound impact on physiology and diseases. 

This work aims to unify, resume and highlight all the main scientific and technological developments concerning DO determination. According to that, two sections can be identified in this article. The first section is an overview of all main oxygen detection methods, each described in terms of basic principles, operation processes, and major advantages, limits, and application fields. These methods also include those conventionally employed in non-biological settings (waters, etc.) but show promise in application to biological matrixes. The second focuses on the applicability of general techniques to DO detection ex vivo and in vivo for clinical applications unitedly to typical in vivo procedures in medical research.

As the reader will see, DO determination is extremely faceted due to the complexity of the matrix studied and the analytical techniques exploited; additionally, this topic interests a range of people with different backgrounds (such as medics, physicists, chemists, and engineers). To achieve a base common knowledge necessary for the full comprehension of the manuscript, the last part of this section will be dedicated to a series of concepts that are probably extraneous to the non-clinical audience. In particular, a brief description of the clinical states associated with DO and the parameters used to quantify and classify those states is reported.

Normal oxygenation levels in human organisms (normoxia, or more accurately, physoxia [5]) depend on the nature of the tissue and are affected by inspiration and expiration phases [6]. Pathological hypoxia is a condition caused by DO levels lower than normal. Since oxygen is tightly coupled to the production of cellular energy, low DO levels cause a decrease in the cellular energy state [7], triggering a vast transcriptional cascade regulating multiple genes [8], which may be associated with pathologies such as ischemia [9] and tumors [10,11].

On the other hand, hyperoxia is associated with higher-than-normal DO levels causing the formation of highly reactive byproducts called ROS (Reactive Oxygen Species) that can react with biological macromolecules causing intracellular damage [12,13,14,15]. 

**Figure 1:**
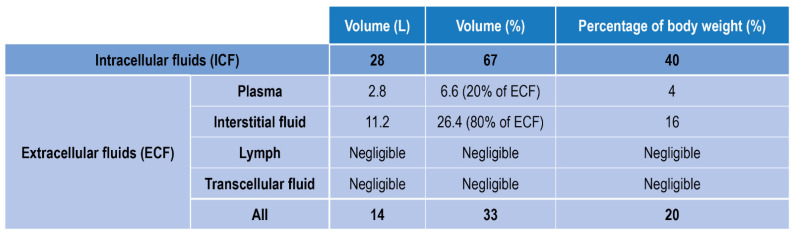
Body fluids classification and relative amount in volume and weight.

Fluids in the human body can be divided into two main classes: fluids within the cells, i.e., intracellular fluids (ICF), and fluids surrounding cells, i.e., extracellular fluid (ECF) (Figure 1). ECF represent 33% of the total human fluids content and includes (1) Plasma, the liquid part of blood, (2) Interstitial fluid, which mediates the interactions between the blood vessel and cells content (3) Lymph (4) Transcellular fluids, (5) cerebrospinal fluid, and to a lesser percentage synovial and pericardial fluid and aqueous humor [16]. 

Another fluid that is a byproduct of the body is urine, and its DO content can also be considered informative, especially when urinary tract infections are considered [17].

Other biological fluids characterized by a high clinical relevance are those used in fluid-based therapies, such as isotonic and hypotonic solutions, enteral fluids, and dialysis and perfusion solutions [18,19,20]. DO levels can be expressed in absolute concentration units (mmol/L, mg/L) or relative units, such as saturation (%, the relative amount of oxygen compared to the maximum amount of oxygen that dissolves in a given liquid at a given temperature). DO levels are typically expressed either as content/concentration (C_O2_, mg/L), as its corresponding partial pressure pO_2_ (in mm Hg or kPa or %), or as Oxygen saturation (S_O2_). The latter expresses the ratio between the actual DO concentration of the sampled fluid and that of an oxygen-saturated solution. C_O2_ in a certain fluid at a certain temperature in saturation conditions is correlated to the partial pressure of O_2_ in equilibrium with the liquid and is described by Henry’s law:

$$C_{X}=k_{x,T}\cdot \mathrm{pX}$$

where C_x_ is the concentration of the dissolved gas x, k_x,T_ is Henry’s law constant, and pX is the partial pressure (in mm Hg or kPa). 

k_x,T_ varies with temperature and liquid composition. An increase in temperature causes a decrease of k_x,T_ thus a decrease in gas solubility. Fluids that naturally (e.g., blood) or artificially (e.g., infusion/perfusion solutions) transport oxygen contain carriers with high O_2_ binding affinity to dramatically raise their O_2_ storage capability. It is important to stress that the carriers do not improve O_2_ solubility in the sample but simply act as O_2_ traps. Consequently, a distinction between solubilized and bound DO is usually made while describing such fluids [21], and the three DO-related parameters (C_O2_, pO_2_, and S_O2_) carry different clinical meanings. For example, for arterial blood:

(1) Oxygen content (Ca_O2_) measures the total oxygen content in arterial blood.

(2) Partial pressure of oxygen (paO_2_) measures the pressure of oxygen dissolved in the arterial blood and how well oxygen can move from the airspace of the lungs into the blood.

(3) Oxygen saturation (Sa_O2_) refers to the percentage of hemoglobin binding sites in red blood cells that are carrying oxygen.

**Figure 2:**
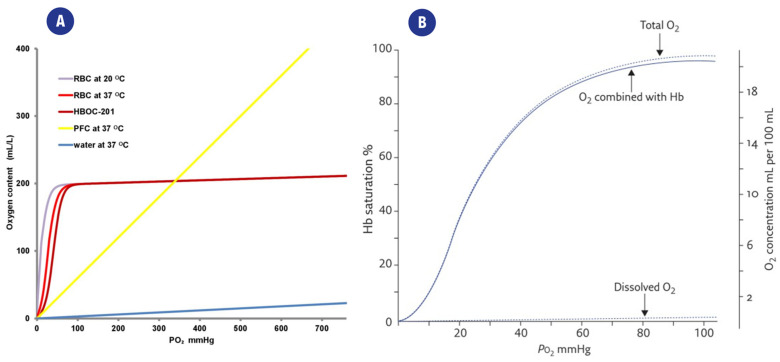
(**A**). Representation of the relation between partial oxygen pressure(pO_2_) and the C_O2_ content of different solutions. O2 is poorly dissolved in water (blue curve), while the presence of carriers such as red blood cells (RBC), perfluorocarbons (PFC), or a hemoglobin-based oxygen carrier (HBOC-201) greatly improves the C_O2_ of the fluid. From [21] (**B**). Typical “dissociation curve” describing the relationship between paO_2_ and Hemoglobin saturation (Sa_O2_). The distribution of total DO in O_2_ combined with heme and dissolved O_2_ is also highlighted. From [26].

The same considerations apply to fluids other than arterial blood: relative or absolute measurements of DO content must be chosen according to clinical and therapeutical goals and their application. The relationship between C_O2_, and pO_2_ is shown in Figure 2a, which also highlights the differences arising from the presence/absence of oxygen carriers.

The relationships between these parameters are quite complex, and a series of mathematical models have been developed to describe them, such as the Equation proposed by Severinghaus to describe the relationship between Sa_O2_ and paO_2_ [22,23]:

Sa_O2_ (%) = ((((paO_2_^3^ + 150 paO_2_)^−1^ × 23,400) + 1)^−1^) × 100

The relationship between paO_2_ and Hemoglobin saturation (Sa_O2_) is also represented in Figure 2b.

This already complex background is further complicated while defining the concentration limits typical of each clinical state since each cell type/tissue has its own physiological parameters. Average values of pO_2_ in tissues generally consider physoxia to range between 22.8–53.2 mmHg (3–7% oxygen), pathological hypoxia is often associated with levels below <15.2 mmHg (2% oxygen) ([5], Table 2), while increases in ROS production due to hyperoxia typically occur for DO levels higher than 100 mmHg (13.5% oxygen). Further information on DO levels in clinical settings can be found in the review by Singer et al. and McKeown et al. [5,24].

The composition (thus also DO levels) of biological fluids also depends on a series of parameters spacing from the area and time of sampling to the health of the patient and the objectives of the clinical field and applications. DO concentration in blood is also greatly affected by the phases of the respiratory cycle [6]. Moreover, pathological conditions such as hemolysis may affect the carrier’s binding properties and, thus, the fluid’s S_O2_ values [25].

Since the change in the dissolved oxygen concentration in a living being is a continuous and dynamic process [5], high-accuracy, rapid, and real-time DO detection methods are essential for in vivo/ex-vivo measurements.

## 2. Classical Methods for DO Monitoring in Biological Fluids

The classical determination methods of DO include titration (Winkler method), optical methods, and electrochemical methods [27,28,29].

### 2.1. Titration Method (Winkler Method)

The classical determination methods of DO include titration (Winkler method), optical methods, and electrochemical methods.

Titration using Winkler analytical procedure is a classical laboratory method for DO determination. The basis of the method relies on iodine ions being quantitatively oxidized to iodine by DO; the amount of iodine generated is determined by titration with a standard thiosulfate solution. The endpoint is determined either by the absorption of ultraviolet light by the tri-iodide ion in the automated method or by using a starch indicator. The amount of oxygen can then be calculated from the titer test: one mole of O_2_ reacts with four moles of thiosulfate [30].

**Figure 3:**
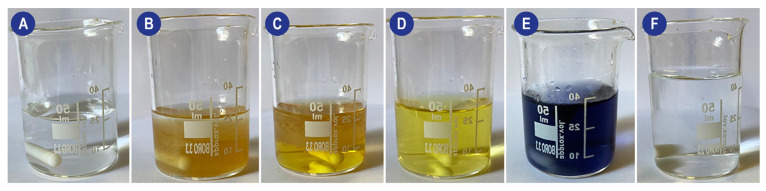
Schematic of the visual changes occurring during a Winkler method-based titration of a perfusion solution. (**A**) Perfusion solution (**B**) Addition of Mn, KI, and KOH (turbid solution with MnO(OH)_2_ precipitate). (**C**) Addition of H_2_SO_4_ (**D**) Characteristic straw yellow color obtained during iodine titration. When reached, the starch indicator must be added to the system. (**E**) System after the addition of the starch indicator. (**F**) System at the end of titration.

The practical experimental procedure involves several steps: (1) A MnSO_4_ and NaOH solution (Reagent I) must be added to the sample in a gas-tight container; this causes the DO to oxidize an equivalent amount of manganese ions to hydroxide (which precipitates). (2) Reagent II (an NaI and H_2_SO_4_ solution) is then added. The acid dissolves the precipitate, and in the presence of iodine ions (I^−^), iodine (I_2_) will be released accordingly to the amount of DO. (3) Finally, the generated iodine is titrated with a thiosulfate solution in the presence of a starch indicator to determine the number of iodine molecules in the solution. The process and the color changes are shown in Figure 3. 

The number of measured iodine molecules is proportional to the number of DO molecules in the original sample, as detailed by the following equations:

     Mn(OH)_2_ + ½ O_2_ → MnO(OH)_2_ (↓)

        MnO(OH)_2_ + 2I^−^ + 4H^+^ → Mn^2+^ + I_2_ + 3H_2_O

I_2_ + 2S_2_O_3_^2−^ → 2I^−^ + S_4_O_6_^2−^

#### Advantages, Disadvantages and Applications

The titration method is highly accurate and precise. On the other hand, this procedure is laborious, time-consuming, and cannot be applied to online measurements [31]*,* and a large sample volume is required for the measurement. Moreover, this procedure is sensitive to contaminants and substances like hydrogen peroxide or nitrite. Finally, the color and turbidity of samples may also cause errors in the measurement [32]. Due to these characteristics, this analytical approach is often used to calibrate other instruments, such as electrochemical electrodes.

Recently an improved version of the method using KIO_3_ as the standard reagent to quantitatively determine the concentration of DO has been proposed. The improved method was fast, with fewer reagents and sufficient accuracy and precision for daily work [29].

### 2.2. Optical Methods

Optical methods can be divided into colorimetric and fluorescence-based methods. 

#### 2.2.1. Colorimetric Methods

The advantages of colorimetric methods (simple chemistry and the possibility of visual detection) are overcome by several disadvantages, such as low resolution, slow response, interferences, and not full reversibility. Compared to the Winkler method, colorimetry methods are less accurate; moreover, they are as complex as the Winkler method and thus cannot meet the requirements of online continuous measurements [33].

#### 2.2.2. Luminescence Methods

Many studies concerning the development of DO sensors based on luminescence are described in the literature. Three main mechanisms are exploited: phosphorescence quenching [34,35,36], near-infrared, and absorption principle [37,38]. Nowadays, most commercialized optical DO sensors are based on fluorescence quenching. After the fluorescent dye absorbs visible or ultraviolet light of a specific wavelength, its electrons gain energy, become excited, and release energy to return to the ground state by emitting fluorescence. Since the collisions between oxygen molecules and excited fluorescent substances interfere with the excitation process of fluorescent substances, the content of oxygen molecules in the samples can be determined according to the fluorescence intensity or the fluorescence lifetime generated at the sensitive interface [39,40]. The principle of fluorescence quenching follows the Stern–Volmer equation [41].

I_0_/I = τ_0_/τ = 1 + K_s-v_ × [O_2_] = 1 + k_q_ × τ_0_ × [O_2_]

**Figure 4:**
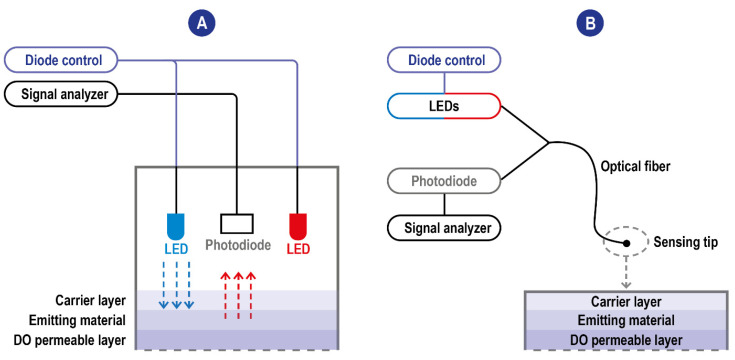
(**A**) Scheme of typical fluorescence quenching-based DO sensors. The blue excitation LED is the one used for the excitation of the emitting material. The red one is used instead as an internal reference. When these sensors exploit the modulation technique, the blue LED is modulated to a frequency related to the luminophore’s luminescence lifetime and the upper and lower lifetimes. Blue arrow: LED emission; red arrow: fluorescence (**B**) Scheme of typical fluorescence quenching-based DO sensors with optic fiber technology. The detector and the light sources communicate are not integrated into the sensing tip and communicate with it through the fiber allowing the miniaturization of the sensing probe.

I_0_ and τ_0_ are unquenched intensity and lifetime at zero O_2_, respectively, I and τ are the corresponding parameters at a given oxygen partial pressure pO_2_, and k_q_ is the quenching constant related to the diffusion rate of oxygen and the luminophore. [O_2_] is the measurement of oxygen concentration. It can be seen from the equation that, for the DO sensor based on the fluorescence quenching principle, the concentration of DO is linearly correlated with the fluorescence intensity. On a structural level, oxygen sensors based on the fluorescence quenching principle are composed of excitation light sources, a DO permeable layer film attached to fluorescence-sensitive substances (the emitting material), and an optoelectronic detection element. The stimulating radiation reaching the emitting material induces a fluorescence response signal whose intensity is measured by a detector (a photodiode). The quenching reaction, reducing the intensity of the response signal, occurs at the interface between the emitting material and the DO permeable layer in the presence of DO when the electrode is put into samples. Most of the time, the parameter measured by the detector is the intensity of the fluorescence radiation; however, sensors exploiting a modulation technique have been developed [42,43]. These sensors are based on the measurement of the phase delay (i.e., a time delay) between the exciting source and the detected red emission from the luminophore, with the phase delay inversely related to the amount of DO [44]. The use of the phase-modulation technique eliminates the impact of intensity fluctuations of the blue LED or the bleaching effects of the luminophore on the measurement. The inverse relationship between DO concentration and phase delay of the emitted red light reduces the signal to allow for better detection of very low DO concentrations. A scheme of typical fluorescence quenching-based DO sensors is detailed in Figure 4.

Fluorescence intensity presents a series of drawbacks: it is affected by many factors, such as power drift of the light source, turbidity, background of the sample’s matrix, and photobleaching of the fluorescent dye [45]. Consequently, it is difficult to construct stable and reproducible sensors based on fluorescence intensity. To solve this problem, sensors based on fluorescence lifetime, an intrinsic parameter of the fluorescence system were developed [42,43].

Aside from the optimal quantification parameter, a key role in improving a sensor’s performance is played by researching innovative materials/species for its components. For example, the ideal matrix on which the fluorescence material layer is deposited must have high oxygen permeability, good mechanical and chemical stability, and excellent optical transparency [46,47].

Nowadays, materials currently used, which satisfy these requirements to an extent, are silicone rubber [48], silica gel [49], sol–gels, and polymers [50,51]. The choice of the fluorescent substance of the sensor influences its performance. These species should be stable, have a high response rate, and should not consume DO. Common substances employed include pyrene, pyrene butyric acid, and fluoranthene, as well as other polycyclic aromatic compounds [52]. To improve detection sensitivity, ruthenium–chromium complexes and platinum phosphor porphyrins can be exploited [53,54].

A significant breakthrough in the DO fluorescence-based sensor is represented by the exploitation of a porous optical fiber as the transmission and detection components of the system first introduced by Peng [55].

Indeed, due to its small size and low weight, an optical fiber allows easy sensor miniaturization [56]. Moreover, it has the advantages of high sensitivity [57], anti-electromagnetic interference ability, and good electrical insulation; it is also relatively easy to use existing optical communication technology to form a telemetry network.

The main DO fluorescence-based oxygen sensors developed are summarized in Table 1; as imaginable due to their superior properties, most of them use optical fiber.

### 2.3. Electrochemical Methods

Electrochemical DO sensors are now the most widely used sensors since they can perform in situ and online measurements [66]. They can be based on conductivity, potentiometry, or current intensity based on their output signal. Intensity-based sensors, which are the most interesting for DO measurement, can also be divided into polarographic and galvanic types. Potentiometric DO sensors contain an oxygen-sensitive material fixed on the surface of the working electrode [67]. When oxygen molecules are close to the sensitive surface, the working electrode is polarized. The voltage difference between the working electrode and the reference electrode is directly proportional to the logarithm of the concentration of DO, thus allowing its quantification [68]. The conductivity-based methods instead exploit selective reactions of compounds (such as thallium) with DO generating ions. The change in conductivity of the solution can be correlated to the DO amount in the sample. Some variants involve a conductimetric titration of the products of the first reaction [30], where DO sensors use thallium or other compounds to react with the oxygen molecules in water to generate thallium ions. Since the chemical reaction on which the sensor is based is specific to oxygen molecules, the concentration of DO can be calculated by measuring the changes in the conductivity of water samples. 

#### 2.3.1. Polarographic Type Electrodes

**Figure 5:**
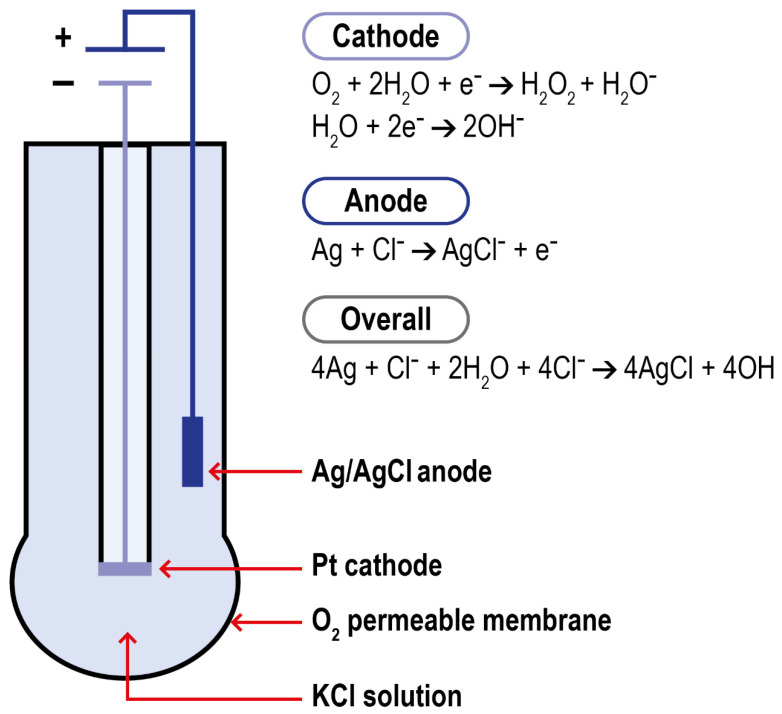
Schematization of Clark electrode and of the reactions in the system.

Polarography is a method for determining the concentration of substances in solution by measuring the current–potential (or potential–time) curve of polarized electrodes during electrolysis. Modern DO polarographic electrodes are composed of a working and an auxiliary electrode (connected by a wire), an intracellular electrolyte, and an air-permeable film (which protects the probe from the sample matrix). This structure was first introduced by Clark in 1956. Clark’s electrode is characterized by a platinum cathode electrode inside an insulating structure on which the anode, an Ag/AgCl electrode, is wrapped around. This system is housed in a plastic cylinder containing a KCl solution which communicates to the external matrix of analysis thanks to a polymeric oxygen-permeable membrane. When the anode of the electrode is polarized by an external power supply, oxygen molecules are reduced on the working electrode, allowing more oxygen atoms to pass through the selective air-permeable film, thus forming a diffusion current. When the correct polarization voltage is selected for a particular electrode, the current output is linear with respect to DO concentration. The electrode setup and the reactions which take place at the surface of the electrode are summarized in Figure 5.

In this context, research is mainly focused on innovative materials able to improve the electron transfer efficiency between oxygen and the electrode surface [69,70,71].

In the process of exploring high-sensitivity materials, the electrochemical principle of the sensor was discovered [72], leading to the development of ECL DO sensors. ECL sensors are characterized by high sensitivity, good selectivity, and good repeatability; moreover, they are easy to control [73].

#### 2.3.2. Galvanic Type Electrodes

The structures of galvanic cells and polarographic sensors are similar. The measurement principle involves an electrolyte-soluble metal anode and an insoluble metal cathode that are immersed in the electrolyte. As the metal of the anode dissolves and oxidizes, it releases electrons that reach the cathode. In the cathode, the oxygen penetrating the thin membrane film acquires these electrons. The current obtained is proportional to the oxygen concentration penetrating the membrane film. Compared to polarographic sensors, galvanic ones are characterized by lower precision, lower output current intensity, and shorter lifetime (related to the wear of the materials during the redox reactions). However, they are characterized by shorter response time and, since the reaction occurs spontaneously, this sensor type requires no power source (thus is easier to use outside a lab). Both sensor classes experience disturbances by chlorine, sulfur dioxide, iodine, bromine, and electromagnetic interference [30].

Furthermore, all electrochemical sensors use an electrolyte solution as a conductive medium which inherently reduces the stability and durability of the sensors and obstacles to their miniaturization. In recent years new solid-state electrochemical sensors adopting a solid material as an electrolyte have been developed. They are characterized by higher stability and durability, and are easier to miniaturize [74,75,76].

Finally, it is worth noticing that aside from miniaturization and research on optimal constructive materials, another important area of study for improving sensors performance (both electrochemical and optical) is represented by the application of technologies for intelligent signal transfer processing, digital signal processing, and real-time dynamic adaptive compensation and correction of DO sensors signals [30]. A comparison of performances of classical DO sensing methods is provided in Table 2.

## 3. DO Detection in Biological Fluids and Physiological Environment 

**Figure 6:**
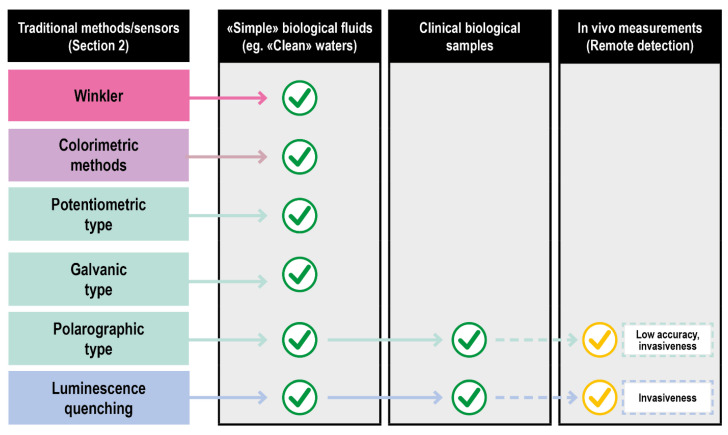
Translation of DO sensing methods from classical to clinical settings of increasing complexity.

Biological fluids of clinical interest comprise human fluids and a series of solutions exploited in fluid-based therapies. Compared to other fluids where oxygen is routinely measured (e.g.*,* natural basins waters), they are characterized by an overall higher chemical complexity and tendency to deteriorate. Consequently, only a few classical approaches can be applied. Titration (Winkler method) suffers from the effects of the interferences and the high delay between the sampling and the measurements. The absorption and emission spectra of oxygen are poorly specific and difficult to assess in a biological context, further invalidating some of the optical procedures. Even the vastly explored electrochemical methods encounter problems in their effective application, such as the difficulty of probing large surface areas and the inherently disruptive nature of the measurement process. In particular, only Galvanic type and fluorescence quenching electrodes showed a reasonable grade of applicability for ex vivo measurements in their miniaturized/optical fiber-based versions as schematized in Figure 6.

A very important feature in the clinical field is the quantification of the analytes in their physiological settings (in vivo measurements). 

To be applied in vivo, the ideal DO evaluation method should possess these additional characteristics: (1) Evaluation of oxygen should be fast, not invasive, and able to monitor multiple points in real-time (at least three sampling points). (2) The sensing equipment should be able to work at different temperatures (4 °C to 37 °C) and fluids. (3) The procedure should not require sampling. Furthermore, large oxygen concentration gradients are believed to exist within even a single organ or granular structure, requiring the ability to quantify DO at different levels (from tissue to subcellular) [6]. For these reasons, titration does not fulfill the requirements and can only be viewed as a suitable tool to calibrate more specific devices prior to application.

The subsections of this paragraph are going to be focused on how the classical analytical methodologies have been translated to the clinical field and on innovative imaging methodologies developed solely to solve the in vivo analytical problem.

### 3.1. Translation of Classical Methods to Clinical Settings: Optical Methods

Optical methods for detecting DO in a biological environment can be divided into two classes based on their founding principle. One exploits the spectroscopic differences between free and heme-bound DO, mostly used to evaluate oxygen saturation (S_O2_). The main techniques belonging to this class are pulse oximetry, diffuse optical spectroscopy and tomography [77], photoacoustic tomography [78], and optical coherence tomography [79]. The second one, described in Section 2.2.2, is based on luminescence quenching caused by oxygen.

#### 3.1.1. Class 1

The pulse oximeter is a particular kind of optical DO sensor widely used in a variety of clinical settings, including emergency and critical care, and is now often part of standard patient observations. It plays a role in monitoring and treating respiratory dysfunction by detecting hypoxemia and is effective in guiding oxygen therapy in adult and pediatric populations [80]. The principle of operation of the pulse oximeter is based on the different light absorption characteristics of hemoglobin at different wavelengths. The absorption spectra of oxygenated and deoxygenated hemoglobin are sufficiently different, so that the distinction can be made with photometric techniques. The system works by transmitting and detecting the differential absorption of two wavelengths of light, typically 660 and 940 nm, through thin tissues, such as a fingertip or earlobe; 660 nm light experiences greater absorption by deoxyhemoglobin, whereas 940 nm light is more strongly absorbed by oxyhemoglobin. By measuring the periodic modulation of this differential absorption, due to pulsed blood flow, pulse oximetry isolates the oxygen saturation of arterial blood alone without contributions from other absorbing species, such as venous blood [6]. The wide increasing use of this sensor is due to a series of positive properties, such as non-invasiveness, portability, effectiveness, and the fact that no exogenous contrast agent is required. The main disadvantage is represented by its limited sensing depth, which makes the resulting saturation oxygen value (Sp_O2_) less accurate than the one obtained through blood gas analysis of arterial blood (Sa_O2_). Although less accurate than Sa_O2_, the typical difference of <2% is usually of no clinical significance [26]. However, pulse oximetry is solely a measure of oxygen saturation (relative, not absolute, amount of oxygen in blood) and gives no indication about blood, pH, carbon dioxide, or bicarbonate concentrations which are useful for patients affected by pathologies such as chronic obstructive pulmonary disease (COPD) or suspected diabetic ketoacidosis [81]. While it is not a substitute for arterial blood gas analysis (Section 3.3) and oxygen absolute concentration determination, overall pulse oximetry can suffice as a monitoring technique when patients are not affected by the risk of respiratory failure or metabolic acidosis pulmonary diseases.

Diffuse optical spectroscopy and tomography (DOS/T) are based on the detection of scattered photons derived from the impact of a series of wavelengths into the tissue. Based on the working mode of the detectors (reflectance or transillumination), their distances from the tissue, and the wavelength used, it is possible to obtain 3D maps, both static and dynamic [82], of tissue parameters, including DO. 

Photoacoustic tomography (PAT) can visualize the three-dimensional position of molecules in tissues by exploiting the ultrasonic wave generated by the expansion of molecules caused by a short burst of photons derived from the tissue. Optical coherence tomography (OCT) instead uses low-coherence interferometry to create high-resolution (<1 mm) tomograms of tissue. Based on the wavelengths and detector used, it is possible to distinguish two different approaches, spectral domain OCT [83] and photothermal OCT [84].

#### 3.1.2. Class 2

Techniques based on S_O2_ determination, though useful for measuring the oxygen contained in blood, rely on the existence of perfusion and do not reveal information regarding the concentration of DO within tissues and cells themselves. Optical-imaging approaches based on luminescence quenching instead enable the direct measurement and quantification of oxygen concentrations within tissues, even in the absence of blood. The principles, advantages, and disadvantages of these techniques have already been discussed in Section 2.2.2. The chemical sensors illustrated up to now represent the most common devices used for DO determination in a variety of sectors (such as food, industrial, and agricultural production). Their application to biological samples is possible and widely explored [56,59]. Real-time in vitro measurement of oxygen uptake rates for HEPG2 liver cells encapsulated in alginate matrices. Their application for in vivo measurements on humans is limited by the invasiveness of the methods, the relatively high oxygen consumption, and the difficulties of mapping large areas. To solve these problems, a series of films, foil sensors and fluorescence quenching imaging probes have been developed [6].

### 3.2. Translation of Classical Methods to Clinical Settings: Luminescence Quenching Imaging Probes

These methods are based on probes that, through incubation or injection enter the sample (cellular culture, animal tissues, or organs). Both intensity and lifetime measurements have been realized with these probes exploiting spectrometers, cameras, and microscopes, providing measurement capabilities on different spatial and temporal scales [85]. Luminescence-intensity-based approaches are advantageous in their simplicity and can be easily adapted to existing imaging equipment. Although these approaches can be limited by inhomogeneous illumination and a non-uniform distribution of the probe molecules, these challenges can be overcome by introducing an oxygen-independent reference dye that co-localizes with the phosphorescent sensor. Lifetime-based approaches, instead, are independent of excitation intensity, detector sensitivity, and probe concentration and have been realized by either time or frequency-domain methods. Time-domain measurements involve exciting the probe molecules with a temporally short light pulse and recording the decay profile [86]. On the other hand, frequency-domain measurements involve exciting probe molecules with modulated light, with lifetimes determined by measuring the phase shift between the excitation and emission signals [87].

The initial probes exploited were simple Ruthenium [88,89] and Iridium complexes [90,91]. Compared to ruthenium complexes, iridium sensors offer a broader color-tuning potential, enabling the synthesis of probes emitting in the near-infrared (NIR) region for deep tissue oxygen sensing.

Ru and Ir possess a limitation on the number and type of ligands that the central metal can accommodate. To obtain a higher grade of flexibility concerning the complexes’ properties, a series of metalloporphyrin classes were developed over the past few decades containing a variety of peripheral groups that can be readily functionalized [92]. To the best of our knowledge due to regulatory hurdles nowadays, only PpIX-based systems are approved by FDA. In addition to Pt(II)- and Pd(II)-porphyrins, luminescent Ru(II)polypyridyl complexes have also been used for intracellular oxygen measurements [93]. Platinum and palladium complexes, in particular, have shown superior photophysical properties for oxygen sensing applications, such as significantly higher room-temperature phosphorescence quantum yields and longer lifetimes than Ru and Ir sensors [94].

The application of these porphyrin-based sensors is limited by their low solubility in aqueous media and their high degree of interactions with the macromolecules present in biological environments. To overcome these challenges, porphyrin molecules have been synthesized with multiple surface functional groups to enable the construction of dendrimers and macromolecular sensors. These constructs can have improved solubilities and biocompatibilities over “naked” porphyrins and can be combined with targeting moieties for improved biodistribution [95,96]. Porphyrin dendrimers structures are well suited for intravascular DO imaging but struggle to pass cells membrane. The first probe of this kind spontaneously able to penetrate multiple cell layers was presented by Nichols et al. [97]. Developments in this field were represented by the introduction of probes that combined the phosphorescence quenching with two-photon microscopies allowing higher dept of visible light excitation [98,99] (a common limitation of these probes). However, the complex multichromophoric structure of porphyrin dendrimers probes is associated with a series of problems nowadays only partially resolved [100], such as (1) generation of alternative quenching patterns caused by the chromophores vigilance, which reduces the probe’s emissivity; (2) emission bands of the original probes usually limiting imaging to no deeper than 300 mm below the tissue surface. [101].

A relevant area of research in this field revolves around the development of new materials to exploit as probes, such as semiconductor nanocrystals [102,103]. In particular, Li et al. 2018 developed a sensor based only on these kinds of materials [104]. MOFs represent another promising class of species that can be used as probes. MOFs with reasonable crystallinity and structural tunability are highly porous, thus able to accommodate high loadings of imaging agents and allow fast diffusion within them. Second, NMOFs are biodegradable due to their relatively labile metal−ligand bond [105]. Lin et al. designed the first fully NMOF-based O_2_ sensor [106]; other systems exploit MOFs just as structural support to the actual sensitive elements due to their outstanding properties [107]. An important breakthrough in recent years, able to solve the problems of previous probes, was represented by Oxyphor 2P developed by Tatiana V. Esipova et al., which was exploited to study DO levels in mouse brains affected by micro strokes [92]. This probe, characterized by a single chromophore structure, unprecedentedly high phosphorescence quantum yield, and high tissue dept accessibility represents the brightest probe developed. In particular, it allows oxygen monitoring twice as deep (up to 600 mm below the tissue surface) and with nearly 60 times higher speed than previously possible.

An important aspect concerning the use of all the probes mentioned is represented by how the probe enters the biological sample of interest. The use of bare probes is not optimal due to solubility and permeability issues and the nontrivial interactions of the probes with the biomolecules of the biological environment. Moreover, the route of endogenous generation of the probes [108,109] most of the time is not applicable. Consequently, the two main strategies used to administer the probes revolver around their conjugation to HSA, PEG [110,111,112], a series of cell-penetrating peptides [113,114,115], or their encapsulation within nanoparticles. Good encapsulating nanoparticles should provide a good layer of protection for the nanoparticles while allowing an easy diffusion of the environmental DO near the probe. Therefore, the porosity of these nanoparticles is a very important parameter. Other two important properties of the encapsulating agents are their loading capability and the ability to functionalize their surface. A high loading capability improves the punctual response of the probes. Cell-uptake studies [116] showed that the lack of a surface cell-targeting layer could result in inefficient cell internalization or increased toxicity; thus, an easy surface functionalization can help to solve these problems. Some of the most common materials used for these nanoparticles are silica [117] and polyacrylamide [118] nanoparticles. Roussakis et al. summarized most of the research on these kinds of systems [6]. The most recent efforts in this field have seen the use of a PMMA-MA and poly(lactic-co-glycolic acid) (PLGA) matrix [119], MOFs [107,120], and semiconducting polymers [121].

**Figure 7:**
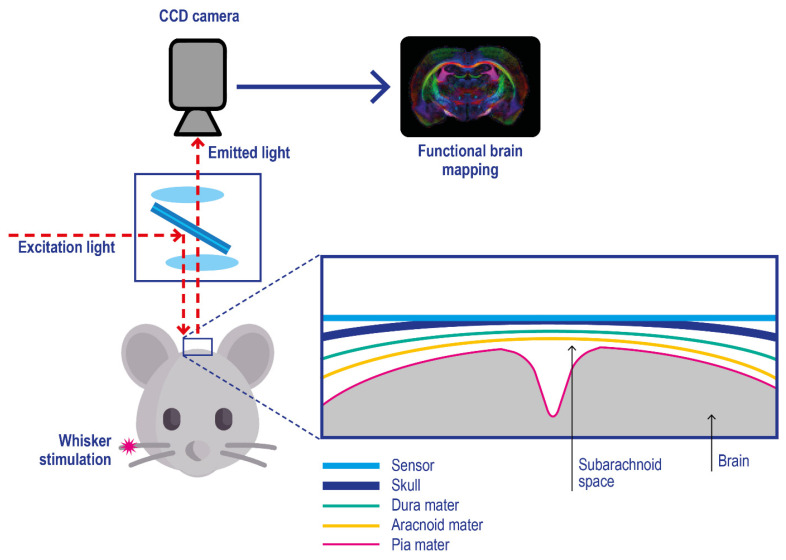
Experimental setup for in vivo optical imaging of oxygen metabolism in brain tissues using a planar phosphorescence PtBP-based oxygen sensor. Pink asterisk: physical stimulation of the rat whisker. The skull cortex of the mouse is illuminated with light at 630 nm, and the emitted photons with a wavelength above 690 nm are acquired with a CCD camera. Adapted with permission from [123].

Film and foil sensors. Film and foil sensors were introduced by Wolfbeis and co-workers [122] to monitor oxygen gradient in engineered tissues. They are typically characterized by a phosphorescent thin-film polymeric-coating applied on an oxygen-impermeable substrate (e.g., polyester films) which is applied onto the target, e.g. brain tissue (Figure 7) [123]; after their application on the target skin, imaging is performed with a CCD camera [124,125].

Starting from this concept, other classes of “surface applicable” sensors have been developed, such as 3D porous scaffolds for cell culture DO monitoring [126,127] and other suspensions of sensor microparticle beads applied on tissue surfaces [128]. All these sensors can only report on O_2_ levels at the tissue surface in contact with the sensor; compared with the probes discussed before, they are characterized by no-invasiveness, making them very promising for in-vivo studies on living beings. They are also reusable. Their application on in vivo pO_2_ imaging allowed them to observe tourniquet-induced forearm ischemia [129], monitor wound surface pO_2_ during healing at split-thickness skin graft donor sites that served as wound models [130] and other 2D oxygen mapping studies [131,132]. A series of dual parameters, such as pH/pO_2_ [125] and T/ pO_2_ [133] sensing films, have been developed, further advancing the research in this field.

### 3.3. Translation of Classical Methods to Clinical Settings: Electrochemical Methods

**Figure 8:**
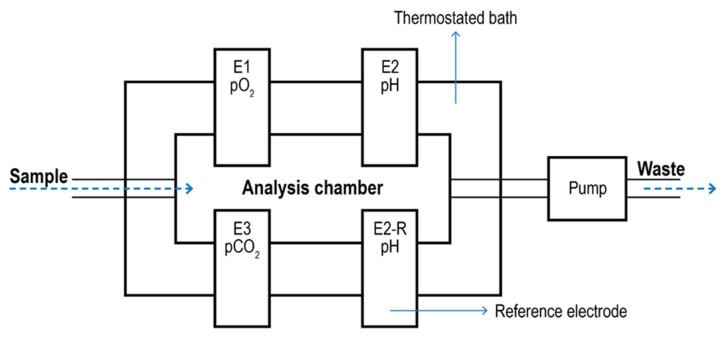
Schematic of a blood gas analyzer system. The sample enters a thermostatic chamber containing the electrodes (E1) due to pump aspiration. E2-R2 represents the pH reference electrode.

Blood Gas Analyzer. Blood Gas Analyzers (BGA) are typical commercial devices based on polarographic electrodes with extensive use in the biomedical field [134,135]. They are mainly used to quantify oxygen and carbon dioxide in the blood. However, some models can also determine the acidity of the blood and the presence of electrolytes (Na^+^, K^+^, Ca^2+^; Cl^-^) and metabolites (glucose, lactate, and total bilirubin). These measurements are typically used to evaluate lung function and acid-base imbalance, which may cause kidney failure, heart failure, and severe infections. In most cases, blood is taken from an artery (Radial, femoral, brachial). The analyzer aspirates the blood into a measuring chamber with Ion Selective Electrodes. In the pO_2_ electrode, oxygen permeates a polypropylene membrane and reacts chemically with a phosphate buffer. The O_2_ combines with water in the buffer, producing a current in proportion to the number of oxygen molecules. The current is measured and expressed as the partial pressure of oxygen. The pCO_2_ electrode is a pH electrode with a Teflon or silicone rubber CO_2_ semi-permeable membrane covering the tip. CO_2_ combines with H_2_O in the space between the membrane and the electrode tip to produce free hydrogen ions in proportion to the partial pressure of CO_2_. The voltmeter, although measuring [H^+^], is calibrated in pCO_2_. The pH electrode compares a potential developed at the electrode tip with a reference potential; the resulting voltage is proportional to the concentration of hydrogen ions [H^+^]. A schematic of a BGA system is given in Figure 8.

The pO_2_ value of arterial blood is a measure of how well the body can absorb oxygen in the lungs and is used to assess how well the body eliminates carbon dioxide, a by-product of metabolism. Finally, the pH value of blood, serum, or plasma is an indicator of the balance between the blood, renal (kidney), and lung (respiratory) systems and is one of the most tightly controlled parameters in the body. Arterial blood-derived parameters such as CaO_2_ and bicarbonate concentration are calculated upon the values of Hb (and the relative SaO_2_) and pO_2_ parameters. Sa_O2_ is calculated based on the assumption that all measured hemoglobin is normal (oxy- or deoxy-) hemoglobin [136]. Although these devices require invasive sampling, the amount of blood required is very low (50*–*95 µL), reducing the overall invasiveness of the technique. From a theoretical stand, since these systems are based on simple polarographic electrodes, they could be used to analyze other fluids (with or without carries) aside from blood to pO_2_, pCO_2_, and pH. Since the relationship between pO_2_, pCO_2_, and pH could be affected by the target medium, specific electrode calibration for the new study matrix must be done. Moreover, new tailormade (liquid-specific) models for the calculation of S_O2_ may be required. To the best of our knowledge, nowadays FDA admits only pleural fluid testing on a blood gas analyzer (and only for pH) [137]. 

DO measurements in tissues. The use of classical polarographic electrodes in tissues is limited by a series of factors such as (1) the invasiveness of the needle-based probes [138]; (2) results highly dependent on measurement location within the tissue; (3) the electrodes require a specific calibration based on the tissue of interest [139]; (4) the needle itself is slightly invasive and potentially damages the tissue [4]. Furthermore, as the probe itself consumes oxygen during point measurements, taking repeated readings at a single tissue location can pose a challenge.

**Figure 9:**
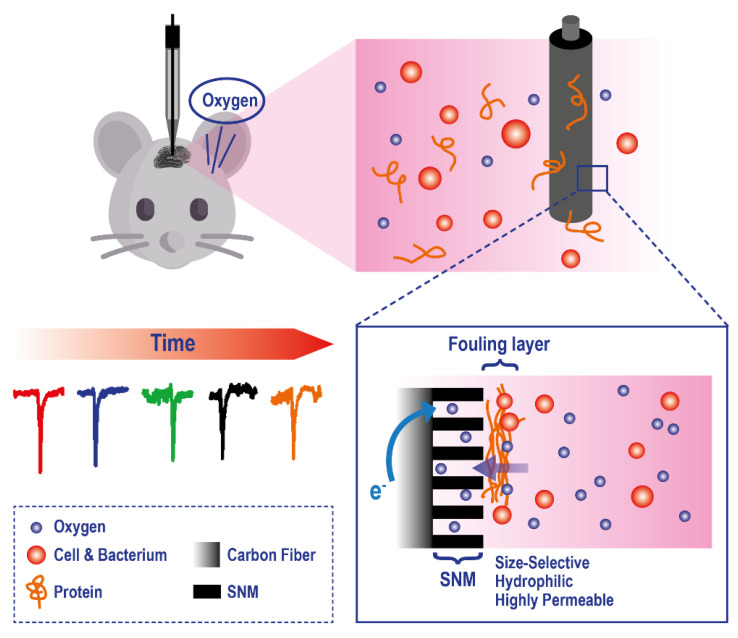
Example of a microelectrode exploiting a Silica and Gold Nanochannel Membrane to monitor in vivo O_2_ levels on rat brain. Adapted with permission from [142].

To improve the applicability of electrodes to this analytical problem, a series of technological modifications have been made. For example, to reduce tissue damage and microcirculatory disturbance, recessed tip microelectrodes have been developed [140,141,142] and applied to in-vivo measurement of O_2_, in organs and tissues, such as rat brain (Figure 9).

There is also a class of planar electrodes that allows the non-invasive transcutaneous measurement of oxygen pressure; however, they require elevated skin temperature of 44*–*45 °C. These technologies cannot map oxygen gradients across the sample; to solve this key problem, new planar polarographic electrodes are in development[143]. Frontiers in this field are represented by the application of new generations of electrochemical sensors-based scaffolds for 3D cell culture models [144] based on what has already been widely explored in the field of luminescence sensors. For example, Weltin and coworkers used electrochemical microsensors to measure metabolic activity from hepatocyte spheroids allowing continuous long-term monitoring of metabolites in a precise manner [145].

### 3.4. Translation of Classical Methods to Clinical Settings: DO Sensing Techniques in Biomedicine

Apart from the translation of conventional methods to clinical settings (optical and electrochemical methods), there are a few techniques that are employed in DO sensing to monitor tissue health and perfusion changes. The first class of methods exploits measuring the presence of radioisotopes and resonance, requiring a contrast medium and expensive instrumentation. Though presenting some interesting features, they are currently not routinely employed for human patients.

**Figure 10:**
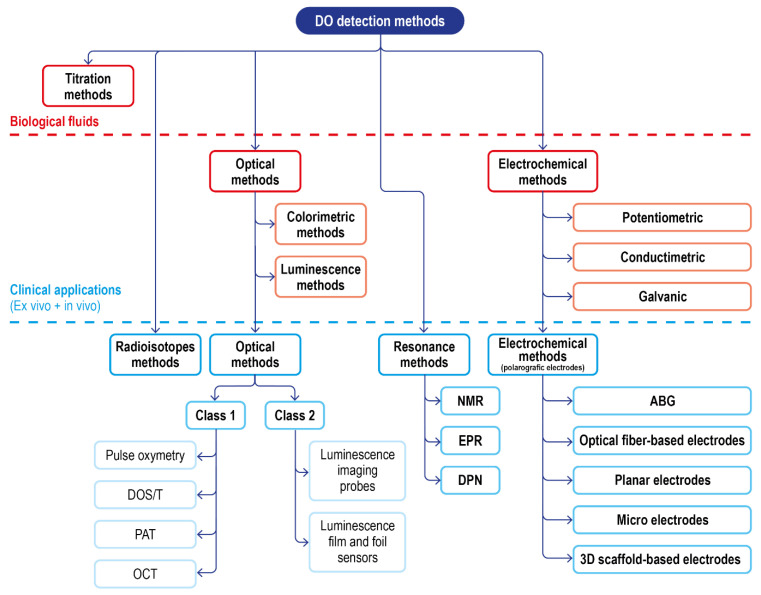
Schematic of the classical DO detection methods in biological fluids and of the advanced solution for clinical applications.

Including these, a summary scheme of the available techniques for DO sensing, and their translatability to DO measurement in a clinical setting, is reported in Figure 10.

#### 3.4.1. Radioisotopes Techniques

These techniques are not invasive and can generate 3D imaging and allow the observation of perfusion changes, oxygen metabolism rates, and the pO_2_ levels of healthy and diseased tissues [146]. Whole-body oxygen imaging can be performed with positron emission tomography (PET) through ^15^O_2_ gas inhalation [147]. Parameters, such as the cerebral blood volume (CBV), cerebral blood flow (CBF), oxygen extraction fraction (OEF), and cerebral metabolic rate of oxygen (CMRO2), can be calculated based on the clearance rate of ^15^O_2_ allowing studies on arterial occlusion, stroke [148], brain tumors [149], and traumatic brain injuries [150].

An alternative to PET is represented by Single Photon Emission Computed Tomography (SPECT). SPECT images are much simpler, less costly to acquire, and can use a wide range of marker isotopes; however, the images are characterized by lower spatial resolution compared to PET. Using this technique, a series of studies, for example, measuring regional cerebral blood flow [151], the effect of hyperbaric oxygen therapy [152,153]*,* and physiological disorders and therapies related to perfusion and oxygen-metabolism abnormalities [154] have been performed. 

Though highly promising, the clinical translation of radioisotope techniques as a routine procedure for tissue oxygen imaging is blocked by the complexity of the measurements, the exposure of patients to radiation, a low spatial resolution, and the need for the on-site production of certain radioactive tracers. Moreover, measurements can only be performed within perfused tissue regions and do not directly provide pO_2_ values (no direct measurement). However, PET continues to be the main technique for clinical imaging of brain circulation

#### 3.4.2. Resonance Techniques

Resonance techniques such as electron paramagnetic resonance (EPR) and dynamic nuclear polarization (DNP) have been exploited for DO imaging. Compared to radioisotope techniques, the contrast agents used do not have to possess a short half-life and thus can be stored up for a long period of time; moreover, the magnetic field used is completely non-invasive. In the past few decades, these methodologies have enabled the non-invasive 3D full-body imaging of a variety of physiologically relevant parameters and have significantly improved our understanding of oxygen distribution on the level of tissues and organs [155,156]. However, they rely on advanced instruments that are large and expensive, which renders their use difficult in a number of clinical and field scenarios; moreover, the imaging resolution is lower compared to radioisotope imaging limiting their capability to resolve microscopic tissue information. Chemometric methods have been applied to mitigate these imaging resolution problems in dynamic imaging [157]. Nuclear resonance techniques have been developed based on ^19^F and ^1^H MR. The imaging is generated by the excitation of the atomic nuclei of interest with a radiofrequency pulse and the subsequent monitoring of the decay rate of the radiofrequency signal emitted during the relaxation process.

^19^F oximetry utilizes the non-toxic perfluorocarbons (PFCs) as exogenous contrast agents [158,159]*,* where lattice relaxation rates of ^19^F are linearly dependent on oxygen partial pressure. PFCs are administered intravenously in emulsions or nanoparticles several hours to a few days before the measurement [160]. The hydrophobicity of the compounds makes them minimally affected by ions proteins in the bloodstream [161]. The main limitations of the technique are the requirements of calibration of the system under experimental conditions and the need for expensive instrumentation able to perform ^19^F imaging. Both short and long-term oxygen monitoring has been reported on numerous animal models [162]. The localized injection of the contrast agent to obtain an immediate image of a certain area was also shown as a viable pathway [163,164]. The technique was also used to evaluate the effect of intervention correlated to changing oxygen perfusion and metabolism [165].

^1^H MR imaging techniques can exploit both endogenous and exogenous contrast agents. The endogenous method uses hemoglobin (dHb) as an endogenous contrast agent and exploits the BOLD [156] and TOLD [166] effects. The method principle is based on the difference in the magnetic susceptibilities between oxyhemoglobin (oxyHb) and deoxyhemoglobin. Despite being non-invasive, this technique is characterized by problems, such as the lack of direct correlation between the amount of excited state and pO_2_ [167] and the results dependence on blood volume [168]. Although ^1^H endogenous imaging is not particularly good for obtaining an accurate evaluation of pO_2_ in the bloodstream due to its high sensibility, it is exceptionally good at detecting variation in the DO levels [6]. Nowadays, this approach is widely applied in the monitoring of Human cerebral blood oxygenation [156], monitoring of tumor microenvironments [169], and evaluation of tumor oxygenation [170].

The exogenous contrast agent technique (also called PISTOL, proton imaging of siloxanes to map tissue oxygenation levels) revolves around the use of siloxanes as contrast agents, such as hexamethyldisilane (HMDSO) and others [171,172]. Limitations of the technique mainly arise from the intrinsic disadvantages of exogenous contrast agents, such as possible undesired biodistributions, results influenced by variances in the clearance rates of these contrast agents, and the invasiveness associated with their injection, which may be multiple for long-term monitoring. In 2019 Shankar and Kodibagkar developed a faster version of PISTOL (PISTOL-LL) exploiting a siloxane-selective Look-Locker imaging sequence equipped with an echo planar imaging (EPI) readout to improve acquisition time [173].

EPR, electron paramagnetic resonance, is a technique capable of detecting paramagnetic chemical species such as oxygen. However, the biological environment requires the additional use of contrast agents that can be divided into soluble materials (e.g., nitroxides, triaryl methyl radicals) and insoluble particulate materials (e.g., lithium phthalocyanine, coals, chars, inks, and carbon blacks) [168]. The particulate ones are characterized by higher spin densities which provide greater sensitivities[174]; moreover, they are stable in a wider pH range and redox conditions. EPR oximetry has been used to measure oxygenation in a wide range of murine organs [175,176]*,* showing high sensitivity and ability to perform repeated measurements [177,178] for long-term monitoring. In recent years quantitative EPR has been applied to correct and improve ^19^F magnetic resonance results [179]. EPR oximetry in humans has thus far been conducted using probes composed of ink particulates; these probes are limited in that they can only measure oxygen if placed within a few mm of the skin surface [180,181]. The most recent advance in human experimentation revolves around the implantation of a small lithium octa-n-butoxynaphthalocyanine crystals-based chip which improves detection depth up to 1.5 cm [182].

An alternative to EPR, dynamic nuclear polarization (DNP) detects free radicals in biological samples by collecting NMR images while irradiating specific EPR resonances [183].

**Figure 11:**
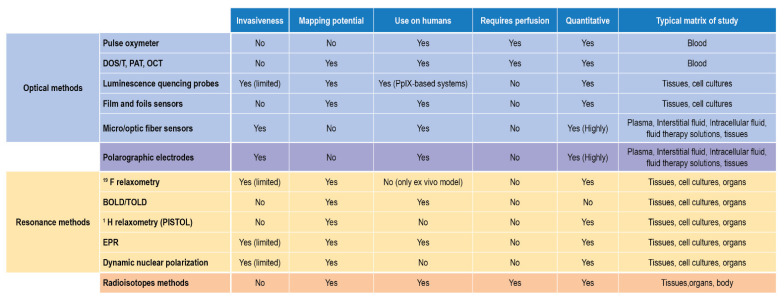
DO detection methods for biological matrices and clinical settings.

In terms of applicability to biological samples and in vivo- ex vivo measurements, these two approaches complete the scenario of suitable techniques, each with some limitations, able to detect, monitor, and quantify DO. Their reported use and their main features are finally summarized in Figure 11.

## 4. Conclusions

Due to the key role dissolved oxygen has in all aspects of life and health, it is easy to understand its importance. Concerning the biomedical field, deviations in DO levels (Hyperoxia and Hypoxia) from homeostasis (Normoxia/Physoxia) influence processes, such as ROS production and metabolism. For instance, hypoxia has been established to play a role in ischemic etiology, it is known to occur in tumors, and it is an important clinical factor in cancer-treatment planning and efficacy, while hyperoxia can occur during therapeutic administration of high concentration or pure oxygen against respiratory failure (such as COVID19 treatment strategy [184]), affecting tissues health and possibly causing ROS-induced damage. 

Oxygen monitoring in a biological setting is very faceted, mainly because of (1) Liquid-specific features, since oxygen content is dependent on the liquid type in which it has to be quantified (salinity, temperature, the composition of the solution can affect DO levels); (2) Patient-related features in vivo, since body Temperature, arterial/venous pressures and health conditions affect the oxygen levels; (3) Oxygen level types of expressions, since oxygen content can be expressed either in absolute or relative measurement units. The type of target expression should be properly chosen, considering the underlying clinical needs and aims of the monitoring; (4) Different sample handling needs based on the location of the source.

Commonly used methods of DO detection include electrochemical detection, optical detection, and iodometric titration. Polarography is currently the most widely used electrochemical method due to its wide applicability; however, it suffers from long polarization time, oxygen consumption, and difficulty in maintenance which reduces its potential for long time measurements. The optical (luminescence) methods overcome the problem of oxygen consumption and have a high sensitivity, fast reaction time, and low maintenance requirements. The development of these sensors revolves around the discovery and implementation of new constructive materials, which allows their miniaturization and better performances (longer life, high sensitivity) without requiring frequent maintenance and calibration. Within this framework, fluorescence sensors seem to be the most promising class, even from a commercial point of view. 

DO determination and monitoring for clinical in vivo analysis represent a more complex problem due to additional requirements. The ideal measurement tool should not require sampling, should not be invasive, and should be able to work on various fluids and temperatures. Moreover, different clinical contexts require different ranges, such as hypoxia (in the pathological state) and hyperoxia (induced by treatment), and monitoring DO in these situations could require additional attention. Last, high DO values in a compartment or fluid (such as perfusion or inhalation therapy leading to high DO levels in the blood) do not necessarily reflect on out-of-scale values in downstream analyses, such as tissues. 

Classical electrochemical and luminescence electrodes have successfully been translated to clinics: the most widespread methods are BGA and pulse oxymetry. However, BGA is an offline measurement, requiring sampling and new calibrations for target liquids, if different from blood, to determine O_2_ absolute concentration. Moreover, it would risk going outside scale for high DO values such as those expected for hyperoxia. Commercial equipment based on these technologies is nowadays used routinary in clinical procedures. The use of optical fibers as probes played a key role in this process allowing remote transmission and high anti-interference abilities and reducing the invasiveness of the sensors. Noninvasive film and foil sensors represent the newest frontier in noninvasive sensor development for oxygen mapping. Dual parameters models (pH/pO_2_; T/pO_2_) exhibited outstanding results; however, they are able to map only a small portion of the body near the skin region where the sensor is attached. Polarographic sensors are interesting since they have a wide application range and can be applied even in vivo, and are able to extend their measuring range outside physiological ones, but consume oxygen during the measurement, which would hinder the evaluation of high DO effects in tissues that could otherwise undergo ROS formation [24].

New strategies have also been developed, such as luminescence, radiometric, and magnetic/electron resonance-based probes. These tools allow the generation of oxygenation maps in humans at the micro and macro levels and provide detailed insight into disease mechanisms and treatment responses. However, they are still subjected to problems that differentiates from class to class, such as the low spatial resolution of the images, the exposure to radiation, the requirement of expensive instrumentation, and low sensibility in detecting areas away from the skin surface. Another relevant challenge is their efficient and targeted delivery, which is particularly difficult for ischemic and cancer regions, which can lie many cell layers away from the blood supply. Although many promising studies have been reported using these probes, their use on human beings is still far from being fully explored due to regulatory hurdles.

At present, there is no method able to completely fulfill all requirements imposed by clinics and sample restrictions; some are more precise, some are faster, and some are more indicated for application in a clinical context. Consequently, the choice of the optimal technique for the clinical field of interest should require the evaluation of the major advantages and disadvantages of each one and the consideration of the specific clinical application of interest and its objectives, the time requirements (continuous, real-time, segmented.), the measurement type (absolute or relative measure) and invasiveness (contactless, immersion), and should also envision the combination of two or more strategies to ensure correct quantification. 

## Figures and Tables

**Table 1:** Summary of available fluorescent oxygen sensors.

Oxygen Indicator	Matrix	Sample Type	Signals	T(°C)	O_2_ Range	Sensor Type	Ref
P(Pt- TPPTFEMA) P(Pt-TPP-EMA)	quartz substrate	cephalosporin C	Luminescence intensity	20–28 °C	0–100% (0–35 ppm)	Optical fiber	[54]
Pd (II) TFPP Pd(II) TCPP Pt(II) TFPP Pt(II) OEP CdSe QDs	sol-gel	aqueous oxygen	Ratiometric Luminescence intensity		0–40 mg/L	Optical fiber	[58]
[Ru-(dpp)_3_]^2+^ Oregon green 488-dextran	sol-gel	Rat C6 Glioma Living Cells	Ratiometric Luminescence intensity	21 °C	0–30 mg/L LOD 7.9 ±2.1 ppm	Silica nanosensor	[59]
PtOEPK OEP	PVC	inter- and intra-cellular	Ratiometric Luminescence intensity	22 ± 0.5 °C	0–43 ppm LOD 19 ppb inter 22 ppb intra	Unpulled PVC fiber sensor	[60]
Ag NPs doped with Ru(DPP)_3_Cl_2_ Coumarin6	PMMA	aqueous oxygen *Chlorella vulgaris*	Ratiometric Luminescence intensity	0–13 mg/L.	Optical sensor LOD = 0.1–0.6 mg/L		[61]
PtTFPP	PDMS pillar arrays	aqueous oxygen enzymatic oxidation of β-d-glucose	Luminescence intensity	23 °C	0.00–1.28 μmol/L LOD = 0.1 μmol/L	Optical sensor	[62]
Ruthenium (II) dichloride (RD3)	silicone layers plus PC-coating	aqueous media (DO in water)	Luminescence intensity	25.0± 0.5°C	LOD = 0.0 4 mg/L	Optical sensor	[63]
Ruthenium complex	Sol-gel	waste-water monitoring	Luminescence lifetime	5–30 °C	(LOD) 6 ppb	optoelectronic sensor	[64]
[Ru(dpp)_3_]^2+^	sol-gel oxides	microenvironments	Luminescence intensity	25°C	IN_2_/IO_2_ from 3 to 35	Optical chemical O_2_ sensors	[65]

**Table 2:** Summary comparison between the three main classical DO detection methods.

	Winkler Method	Polarographic Methods	Fluorescence Methods
**Remote monitoring**	Cannot achieve remote measurement, and samples must be analyzed in the laboratory.	Can achieve remote detection, but the signal transmission will be distorted; thus, the detection results are not accurate.	Can use an optical fiber to transmit signals and achieve remote detection.
**Analysis/response time**	Longest time required	30–180 s; however, the polarization of the electrode requires about 15 additional minutes, so the response time is longer [30].	41 ms-694 s. The fluorescence quenching method has the fastest response time (up to the ms level) [30].
**Oxygen consumption**	Yes (titration process)	Yes (redox reaction at the electrode)	No (quenching process is reversible)
**Maintenance**	No	Yes	No
**Application**	Laboratory and water samples.	Biological medicine, forestry, fishing.	Life sciences, harsh environments.
**Interference**	Turbidity, nitrite, free chlorine, iron ions, colored solution.	Chlorine, sulfur dioxide, Iodine, Bromine, Electrical interference.	Fluorescence quenching and the stability of organic molecules can be influenced by factors that include pH and temperature.
**Accuracy**	±0.1% [30]	±0.01–0.1 mg/L [30]	/

## Data Availability

Not applicable.
